# Quality of life and treatment options in patients with genital psoriasis: The Filipino experience

**DOI:** 10.1016/j.jdin.2025.05.007

**Published:** 2025-06-09

**Authors:** Roy Luister C. Acos, Maria Emilia Ruth V. Eusebio, Krisha K. Lim, Giselle Marie Tioleco-Ver, Ma. Lorna F. Frez

**Affiliations:** a Department of Dermatology, University of the Philippines - Philippine General Hospital, Manila, Metro Manila, Philippines; b Section of Dermatology, Department of Internal Medicine, Adela Serra Ty Memorial Medical Center, Tandag City, Surigao del Sur, Philippines

**Keywords:** dermatology life quality index, genital psoriasis, Philippines, quality of life, sexual function, treatment

*To the Editor:* Genital psoriasis (GenP) is a frequently overlooked yet significant manifestation of psoriasis, causing severe impairment in patients’ quality of life (QoL).^1^, ^2^, ^3^ This study aims to assess the burden of GenP among Filipino adults, emphasizing its prevalence, treatment options, and physician engagement.

A cross-sectional survey was conducted among 461 Filipino psoriasis patients recruited from Psoriasis Philippines and a tertiary hospital outpatient clinic. An email was sent to the Psoriasis Philippines board to disseminate the survey form to its members. Data collection involved self-administered questionnaires evaluating patient demographics, GenP prevalence, treatment patterns, Dermatology Life Quality Index scores, and patient-physician interactions regarding GenP.

Among respondents, 86% reported current or past genital involvement, with 67% experiencing active GenP. The mean age of onset was 29.2 ± 11.1 years (Table I).

**Table I:** Baseline patient characteristics (*n* = 461)

Characteristic	$\bar{x}$/f	%
Sex		
Male	149	32.32%
Female	312	67.68%
Marital status		
Single	198	42.95%
Married/cohabiting	245	53.15%
Others	18	3.90%
Employment status		
Unemployed	160	34.71%
Employed	301	65.29%
Type of psoriasis		
Plaque	244	52.93%
Scalp	101	21.91%
Guttate	85	18.44%
Palmoplantar	3	0.65%
Inverse	5	1.08%
Pustular	12	2.60%
Erythrodermic	11	2.39%
Complications		
With joint complaints	259	56.18%
With nail involvement	328	71.15%
Ever had GenP		
Yes	396	85.90%
No	65	14.10%
Current GenP		
Yes	311	67.46%
No	150	32.54%
Age data		
Age at participation, y∗	37.8 ± 11.1	
Age at onset of psoriasis, y∗	25.9 ± 11.3	
Age at onset of genital psoriasis, y∗	29.2 ± 11.1	

*Current GenP* was defined as involvement of psoriasis on the genital skin including the perianal area in the last 6 months.

*f*, Frequency; *GenP*, genital psoriasis; $\bar{x}$, mean.

∗ Mean ± standard deviation.

Patients with current GenP had significantly higher mean-rank total Dermatology Life Quality Index score (249.96 vs 191.68, *P* value <.0001) as well as all domain scores which indicate a significantly worse QoL compared to patients without current genital involvement (Table II).

**Table II:** Dermatology Life Quality Index (DLQI) scores of the study groups

	With GenP (n = 311)			Without GenP (n = 150)			*P* value
	Median	IQR	Mean-rank	Median	IQR	Mean-rank	
Total DLQI	13	12	249.96	11	10	191.68	<.0001
Personal relations	1	3	244.33	1	2	203.36	.0014
Symptoms and feelings	3	2	247.83	2	2	196.10	.0001
Daily activities	3	3	246.06	2	3	199.78	.0004
Leisure	2	3	246.85	2	3	198.14	.0002
Work and school	0	3	239.59	0	3	213.19	.0179
Treatment	1	1	250.09	1	2	191.42	<.0001

The initial severity of those with GenP history, whether current or not, was not determined, as it was prone to recall bias. Differences in mean-rank total DLQI scores and its domains between the 2 groups were determined using Mann–Whitney U test.

*GenP*, Genital psoriasis; *IQR*, interquartile range.

Topical medications (*n* = 306, 66.4%) emerged as the top choice of treatment, specifically mid-to high-potency topical corticosteroids (eg, mometasone, clobetasol) in 267 patients (58%) followed by methotrexate (*n* = 148, 32.10%) and calcipotriol (*n* = 148, 18.3%). Other topical treatments included coal tar (*n* = 55, 11.93%) and tacrolimus (*n* = 10, 2.17%). Alarmingly, potent steroids were frequently used (58%), raising concerns about inappropriate self-medication and physician prescribing patterns.

Despite the high prevalence, GenP remains inadequately addressed in clinical practice. Less than half of the patients with current GenP (*n* = 139, 44.7%) discussed their genital lesions with their physicians, and only 36% (*n* = 112) underwent a genital examination. Most respondents (*n* = 260, 83.6%) expressed the need for more proactive physician inquiries regarding genital involvement. This highlights a critical gap in clinical communication and patient education regarding GenP management.

The reluctance to discuss genital lesions may stem from cultural stigma, embarrassment, or the perception that GenP is less important than other psoriasis manifestations.^3^^,^^4^ However, given its profound impact on QoL – including personal relationships, daily activities, and psychological well-being – health care workers must take a more proactive role in screening for and addressing GenP.

First-line treatment recommendations suggest the cautious use of low-to mid-potency corticosteroids,^5^ yet this study found a high reliance on potent steroids, which would predispose patients to local adverse effects such as skin atrophy. Increased awareness and patient education regarding safe and effective GenP treatments are essential.

This study has limitations, including its reliance on self-reported data, potential selection bias favoring tech-savvy individuals, likelihood that those with GenP were more inclined to participate due to the study title, and the absence of physician-confirmed diagnoses. Despite the limitations, our findings underscore the high burden of GenP in the Filipino population and the urgent need for improved physician-patient communication and evidence-based treatment approaches.

In conclusion, GenP is a common but under-recognized aspect of psoriasis with a significant impact on QoL. Physicians should actively screen for GenP and educate patients on safe treatment options. Further research is needed to develop culturally sensitive interventions that enhance the management of GenP and improve patient outcomes.

## Conflicts of interest

None disclosed.
